# Editorial: Down the rabbit hole – the psychological and neural mechanisms of psychedelic compounds and their use in treating mental health and medical conditions

**DOI:** 10.3389/fpsyt.2024.1431389

**Published:** 2024-06-10

**Authors:** Leehe Peled-Avron, Jacob S. Aday, Amilia Lydia Kalafateli, Holly K. Hamilton, Joshua D. Woolley

**Affiliations:** ^1^Department of Psychology and Gonda Interdisciplinary Brain Research Center, Bar-Ilan University, Ramat-Gan, Israel; ^2^Department of Psychiatry and Behavioral Sciences, University of California, San Francisco, San Francisco, CA, United States; ^3^Department of Psychiatry, San Francisco Veterans Affairs Medical Center, San Francisco, CA, United States; ^4^Department of Anesthesiology, University of Michigan, Ann Arbor, MI, United States; ^5^NLC Health Ventures, Amsterdam, Netherlands; ^6^Department of Psychiatry & Behavioral Sciences, University of Minnesota, Minneapolis, MN, United States; ^7^Mental Health Service, Minneapolis VA Health Care System, Minneapolis, MN, United States

**Keywords:** mystical experience, entheogen, depression, virtual reality, 5-MeO-DMT, mechanisms

Psychedelic substances have demonstrated promising therapeutic effects across a wide range of mental health conditions including depression (1–13) anxiety disorders including generalized anxiety (14–16) and social anxiety (17, 18) and autism (19), post-traumatic stress disorder (PTSD) (6, 20–23), and substance use disorders such as tobacco addiction (24) and alcoholism (25–30). These therapeutic effects are hypothesized to work through various mechanisms operating at different levels of analysis. At the cellular and molecular level, these include increased stimulation of the 5-HT2A serotonin receptor, increased cortical glutamatergic transmission, increased neuroplasticity, and decreased inflammation (31). At the network and circuit levels, the proposed mechanisms involve decreased brain modularity, changes in network functional connectivity, and increased neural entropy (32, 33). Psychologically and behaviorally, the effects are thought to involve increased cognitive and psychological flexibility, experiences of psychological insight, heightened emotional acceptance, and peak experiences or mystical-type states (34, 35). By further examining the neurobiological and psychological mechanisms of psychedelic substances, we can gain a deeper understanding of how these substances interact with the brain and influence behavior and help alleviate suffering in various psychopathologies.

This Research Topic aimed to highlight new interdisciplinary research on psychedelic substances, furthering our understanding of their mechanisms of action and their potential impact on clinical populations. By bringing together data from basic research, as well as clinical and interventional studies, this endeavor seeks to advance the field and pave the way for the responsible and evidence-based use of psychedelics in the realm of psychiatry and mental health.

To begin, several articles in the Research Topic examined the role of phenomenology as a putative mechanism underlying the therapeutic effects of psychedelics. For example, mystical experiences (i.e., characterized by transcendence of time and space, positive mood, and ineffability) can be reliably induced by psychedelics and have previously been linked to positive therapeutic outcomes across several studies (36). In this Research Topic, Mosurinjohn et al. contribute to the theoretical knowledge in this area by providing an interdisciplinary discussion and critique. The authors note how the discussion of mystical experiences in psychedelic literature has only been minimally informed by decades of research in relevant fields such as religious studies and anthropology. Specifically, by tracing the origins of mystical experiences in psychedelic research, the authors outline how the field has failed to acknowledge its perennialist and Christian bias. Although mystical experiences as currently defined and measured have been a useful construct for the field, the authors demonstrate that stronger interdisciplinary theoretical and empirical approaches are needed.

Further supporting the role of mystical experiences in positive therapeutic outcomes, Ortiz Bernal et al. examined reactivations (i.e., “flashbacks”) after the use of 5-MeO-DMT in a non-clinical sample (N=513). Reactivations constitute a recurrence of specific aspects of the drug-induced experience after the effects of the drug have diminished. Being female, being older at the time of first 5-MeO-DMT dose, having higher educational attainment, and dosing in a structured group setting were all associated with increased probability of reporting a reactivation event. The authors also found that higher mystical experience scores were associated with neutral or positive emotional valence of a reactivation experience. The results suggest that reactivations were commonly reported, but were overwhelmingly perceived as positive or neutral experiences, suggesting they may contribute to therapeutic benefits rather than being adverse effects.

Another alteration in subjective experience that has been linked with psychedelics and may be relevant in long-term outcomes is ayahuasca-induced personal death (APD) experience. During an APD experience, an individual may experience a profoundly convincing sensation of imminent death or being deceased, so realistic that it is indistinguishable from actual death. In their paper delving into reports of ayahuasca users in ceremonies in South America, David et al. examined potential associations between APD experiences and individual characteristics (N=306). Interestingly, no association was found between demographics (age and gender), personality type or psychopathology and these death-like experiences which occurred in 50% of the participants in their sample. Nevertheless, APD experiences were correlated with increased self-reported environmental concern, ability to cope with distressing life events, and the sense of fulfillment in life, suggesting they may contribute to positive outcomes of the psychedelic experience.

Garel et al. also demonstrated how previous environmental stimuli such as exposure to social media, influence the experiences of patients (N=26) undergoing ketamine treatment for treatment-resistant depression (TRD). The authors introduce the concept of “imprinting” to explain time-lagged effects across various hallucinogenic drugs. Findings suggest that higher levels of media exposure prior to treatment reduce mystical/emotional qualities, altering subjective experiences. The study highlights the need to recognize and explore the role of past environmental factors in psychedelic therapy, proposing imprinting as a novel framework to enhance understanding and guide future research efforts.

Indeed, external factors may affect the subjective experience and use of psychedelics, as was explored in Bălăeț et al. during the COVID-19 pandemic. Data was collected between December 2019 and May 2020. Users (N=30,598) who consumed psychedelics and cannabis simultaneously during the pandemic reported decreased mood (over the past month from the time of assessment) compared to users who consumed cannabis without psychedelics or no substances at all. These results shed further light on the contextual and types of substances used to affect mood following psychedelic use.

One somewhat provocative suggestion is that experiencing the subjective perceptual effects related to psychedelics is in and of itself therapeutic. Kaup et al. explored this possibility by developing a virtual reality experience, Psyreal, that mimics the phenomenological components of psychedelic states. In an open-label feasibility study, 13 participants with mild-to-moderate depression underwent a 2-day Psyreal intervention. At the two-week follow-up, the researchers observed a significant decrease in depressive symptoms. Although limited by an open-label study design and small sample size, the results warrant further research on using psychedelic-like experiences induced by virtual reality for the treatment of psychological disorders.

Further substantiating the therapeutic effect of psychedelics on depression, Warner-Schmidt et al. showed that methylone, a rapid-acting entactogen, had a superior antidepressant effect compared to prototypical SSRI fluoxetine in preclinical animal models of antidepressant screening (N=16). Using the forced swim test in adult rats, a single dose of methylone alone reduced immobility by nearly 95% of the animals lasting for 72 hrs post injection compared to SSRI which reduced immobility by 50% lasting for one hour post injection. These results indicate that methylone might have a potential clinical benefit for treatment of depression.

Psychedelics are also beginning to be explored for novel treatment indications, such as attention deficit hyperactivity disorder (ADHD), post-traumatic stress disorder (PTSD), and Fibromyalgia. Haijen et al. explored the effects of microdosing of psilocybin on the behavior of individuals with ADHD in a large cohort of online participants (n=233). They found that trait mindfulness (i.e., description and non-judging of inner experience) was increased and neuroticism was decreased after 4 weeks of microdosing compared to baseline. The authors concluded that microdosing may have potential to induce changes in stable traits and suggest that a larger, controlled study would shed light on the therapeutic effects of microdosing on individuals with ADHD.

Ragnhildstveit et al. presented a case study of cannabis-assisted psychotherapy. They report that a female diagnosed with dissociative-PTSD (D-PTSD), a subtype of posttraumatic stress disorder, no longer met criteria for the disorder following the treatment. The patient experienced a common psychedelic phenomenon during the treatment - ego dissolution. This experience involves a profound loss of self-boundaries, merging with the environment. The experience of ego dissolution facilitated acceptance for this patient, which enabled the patient to access buried emotions and memories related to the trauma. Despite the study being limited to N=1, the case supports further study into cannabis-assisted psychotherapy as a tool for D-PTSD treatment.

Bornemann et al. describes a protocol for investigating the effects of psilocybin -assisted psychotherapy in fibromyalgia patients, aiming to understand its neural mechanisms using electroencephalography (EEG) and functional magnetic resonance imaging (fMRI). The findings aim to deepen the understanding of psilocybin therapy’s neural mechanisms in fibromyalgia, which would benefit future studies on psychedelic therapy.

One paper in this Research Topic looked into the molecular mechanisms that underlie the effects of psychedelics. The study investigated the direct effects of classic psychedelics like LSD, psilocin, DMT, and mescaline on the immune function of human T cells and monocytes *in vitro* (Rudin et al.). Specifically, it examined whether these substances modulated T cell proliferation, cytokine release, and NF-κB induction in monocytes. The results showed no relevant immune-modulatory effects from any of the tested psychedelics on these immune parameters in the cell lines studied. This suggests classic psychedelics do not directly suppress immune function, supporting their potential safe therapeutic use in patients where diminished immunity would be detrimental.

In conclusion, the Research Topic provides an interdisciplinary overview of recent research on the neurobiological and psychological mechanisms of psychedelic substances, highlighting several key findings. Mystical experiences induced by psychedelics are linked to positive therapeutic outcomes but require deeper theoretical examination across disciplines. Contextual factors like prior media exposure and substance combinations significantly influence psychedelic experiences, which should be accounted for in future research and clinical applications. Novel psychedelic substances, as well as non-pharmacological simulations of psychedelic effects, show promise for treating psychiatric conditions like depression. At the same time, psychedelics may also benefit disorders like ADHD, PTSD, and fibromyalgia, though more clinical research is needed. Importantly, *in vitro* studies suggest psychedelics may not have concerning immunosuppressive effects, supporting their safe implementation. Overall, this Research Topic demonstrates the value of an interdisciplinary approach to understanding and responsibly harnessing the complex therapeutic potential of psychedelics in helping individuals to cope and heal from mental illness.

## Author contributions

LP: Writing – original draft, Writing – review & editing. JA: Writing – original draft, Writing – review & editing. AK: Writing – original draft, Writing – review & editing. HH: Writing – review & editing. JW: Writing – review & editing.
